# Oncogenic Human Papillomaviruses Activate the Tumor-Associated Lens Epithelial-Derived Growth Factor (LEDGF) Gene

**DOI:** 10.1371/journal.ppat.1003957

**Published:** 2014-03-06

**Authors:** Jenny Leitz, Miriam Reuschenbach, Claudia Lohrey, Anja Honegger, Rosita Accardi, Massimo Tommasino, Manuel Llano, Magnus von Knebel Doeberitz, Karin Hoppe-Seyler, Felix Hoppe-Seyler

**Affiliations:** 1 Molecular Therapy of Virus-Associated Cancers (F065), Program Infection and Cancer, German Cancer Research Center (DKFZ), Heidelberg, Germany; 2 Department of Applied Tumor Biology, Institute of Pathology, University of Heidelberg, Heidelberg, Germany; 3 International Agency for Research on Cancer, World Health Organization, Lyon, France; 4 Department of Biological Sciences, University of Texas at El Paso, El Paso, Texas, United States of America; University of Wisconsin-Madison, United States of America

## Abstract

The expression of the human papillomavirus (HPV) *E6/E7* oncogenes is crucial for HPV-induced malignant cell transformation. The identification of cellular targets attacked by the HPV oncogenes is critical for our understanding of the molecular mechanisms of HPV-associated carcinogenesis and may open novel therapeutic opportunities. Here, we identify the *Lens Epithelial-Derived Growth Factor (LEDGF)* gene as a novel cellular target gene for the HPV oncogenes. Elevated *LEDGF* expression has been recently linked to human carcinogenesis and can protect tumor cells towards different forms of cellular stress. We show that intracellular *LEDGF* mRNA and protein levels in HPV-positive cancer cells are critically dependent on the maintenance of viral oncogene expression. Ectopic *E6/E7* expression stimulates LEDGF transcription in primary keratinocytes, at least in part via activation of the LEDGF promoter. Repression of endogenous *LEDGF* expression by RNA interference results in an increased sensitivity of HPV-positive cancer cells towards genotoxic agents. Immunohistochemical analyses of cervical tissue specimens reveal a highly significant increase of LEDGF protein levels in HPV-positive lesions compared to histologically normal cervical epithelium. Taken together, these results indicate that the E6/E7-dependent maintenance of intracellular *LEDGF* expression is critical for protecting HPV-positive cancer cells against various forms of cellular stress, including DNA damage. This could support tumor cell survival and contribute to the therapeutic resistance of cervical cancers towards genotoxic treatment strategies in the clinic.

## Author Summary

Specific types of human papillomaviruses (HPVs) are closely linked to the development of malignant tumors, such as cervical cancer. Virtually all cervical cancers contain HPV DNA and the tumorigenic growth behavior of cervical cancer cells is dependent on the activity of two viral oncogenes, called *E6* and *E7*. It is important to study the activities by which the HPV oncogenes can support the growth of tumor cells. This should allow new insights into the molecular mechanisms of virus-induced carcinogenesis and could also be useful for developing novel approaches for cancer therapy. We here show that the HPV oncogenes stimulate and maintain expression of the cellular *LEDGF* gene in HPV-positive cancer cells. Consistently, pre-malignant and malignant lesions of the cervix exhibit significantly increased LEDGF protein levels. *LEDGF* is crucial for the protection of tumor cells against various forms of cellular stress, including DNA damage. *LEDGF* stimulation by the viral oncogenes could be a critical survival mechanism by which HPVs support the growth of cervical cancer cells and provide resistance towards chemo- and radiotherapy in the clinic.

## Introduction

Oncogenic types of human papillomaviruses (HPVs), such as HPV16 and HPV18, are major human carcinogens. They cause cervical carcinoma, the second most common cancer in females worldwide and are closely linked to the development of other malignancies, including a subset of additional anogenital (e.g. anal, vulvar and penile) and oropharyngeal (e.g. tonsillar) cancers [1]. Two viral oncogenes, *E6* and *E7*, are crucial for both the induction and the maintenance of the malignant phenotype of HPV-positive cervical cancer cells, indicating that cervical cancer cells display features of a phenomenon termed “oncogene addiction” [2]. On the basis of many mechanistic studies, the picture emerges that the two HPV oncogenes inactivate crucial tumorsuppressive responses of the cell, such as induction of senescence or apoptosis [3]–[6]. Importantly, at least some of these pathways are not irreversibly deregulated by HPVs. Rather, inhibition of viral E6/E7 activities in HPV-positive cancer cells leads to the reactivation of dormant tumor suppressor pathways and can eventually result in efficient growth arrest, senescence, and/or cell death [7]–[12].

These latter observations are significant for therapeutic considerations since it should be principally possible to revert the malignant phenotype of HPV-positive cancer cells. In general, this could be achieved by therapeutically blocking the *E6/E7* oncogenes or, alternatively, by correcting the cellular pathways which are deregulated by the viral oncogenes. Thus, it is important to delineate critical cellular targets that are affected by viral *E6/E7* oncogene expression and thereby contribute to the malignant phenotype of HPV-positive cancer cells.

In order to search for cellular genes targeted by the viral *E6/E7* oncogenes, we silenced endogenous HPV18 E6/E7 expression in HeLa cervical carcinoma cells by RNA interference (RNAi) and performed a genomewide transcriptome analysis. Data from this array suggested that the expression of the “*Lens Epithelial-Derived Growth Factor/p75 (LEDGF)*” gene (alternatively called *PSIP1*) is reduced upon *E6/E7* repression [13]. Its major splice product codes for the 530-amino acid LEDGF/p75 protein (in the following called LEDGF), a chromatin-associated factor that is best known for its important role during the human immunodeficiency virus-1 (HIV-1) life cycle. In this context, LEDGF interacts with the viral integrase (IN) and directs integration of the HIV-1 genome into the host cell chromosome [14]–[17].

More recently, however, there is emerging data indicating that *LEDGF* could also play an important role for human carcinogenesis. This notion is supported by the observations that: (i) *LEDGF* is overexpressed in several human cancers when compared with corresponding normal tissue [18]–[20]; (ii) the *LEDGF* gene is a target for chromosomal translocations in leukemias, leading to LEDGF/NUP98 fusion proteins [21] that protect leukemia cells against cell death [22]; (iii) the LEDGF protein contributes to leukemogenesis by tethering the mixed-lineage leukemia (MLL1) complex to cancer-associated target genes [23]; (iv) ectopically overexpressed *LEDGF* increases the tumorigenicity of different cancer cell lines *in vivo* [19], [24], [25]; (v) *LEDGF* can enhance angiogenesis and lymphangiogenesis, thereby possibly contributing to cancer metastasis [24], [26]; (vi) LEDGF can act as a survival factor in tumor cells towards different forms of cellular stress [22], [27]–[32], and (vii) LEDGF plays an important role for the repair of DNA damage [33], consistent with its genoprotective potential [19], [22], [33], [34].

Here, we investigated the connection between HPV *E6/E7* oncogene and *LEDGF* expression, analyzed the contribution of LEDGF to the growth and to the DNA damage response of HPV-positive cancer cells, and examined the *in vivo* expression of the LEDGF protein in biopsies from premalignant lesions and cervical cancer. We show that (i) the maintenance of intracellular LEDGF amounts in HPV-positive tumor cells is critically dependent on continuous HPV *E6/E7* expression, (ii) HPVs can transcriptionally stimulate *LEDGF* gene expression via *LEDGF* promoter activation, (iii) LEDGF is crucial for the growth and survival of HPV-positive cancer cells following DNA damage, and (iv) LEDGF levels are significantly elevated in cervical dysplasias and cancers. We propose that the *E6/E7*-dependent intracellular *LEDGF* expression could be an important determinant for the survival of HPV-positive cancer cells under different forms of cellular stress and for their resistance towards radio-and chemotherapy.

## Results

### Silencing of endogenous E6/E7 oncogene expression in HPV-positive cancer cells leads to LEDGF repression

**Figure 1:**
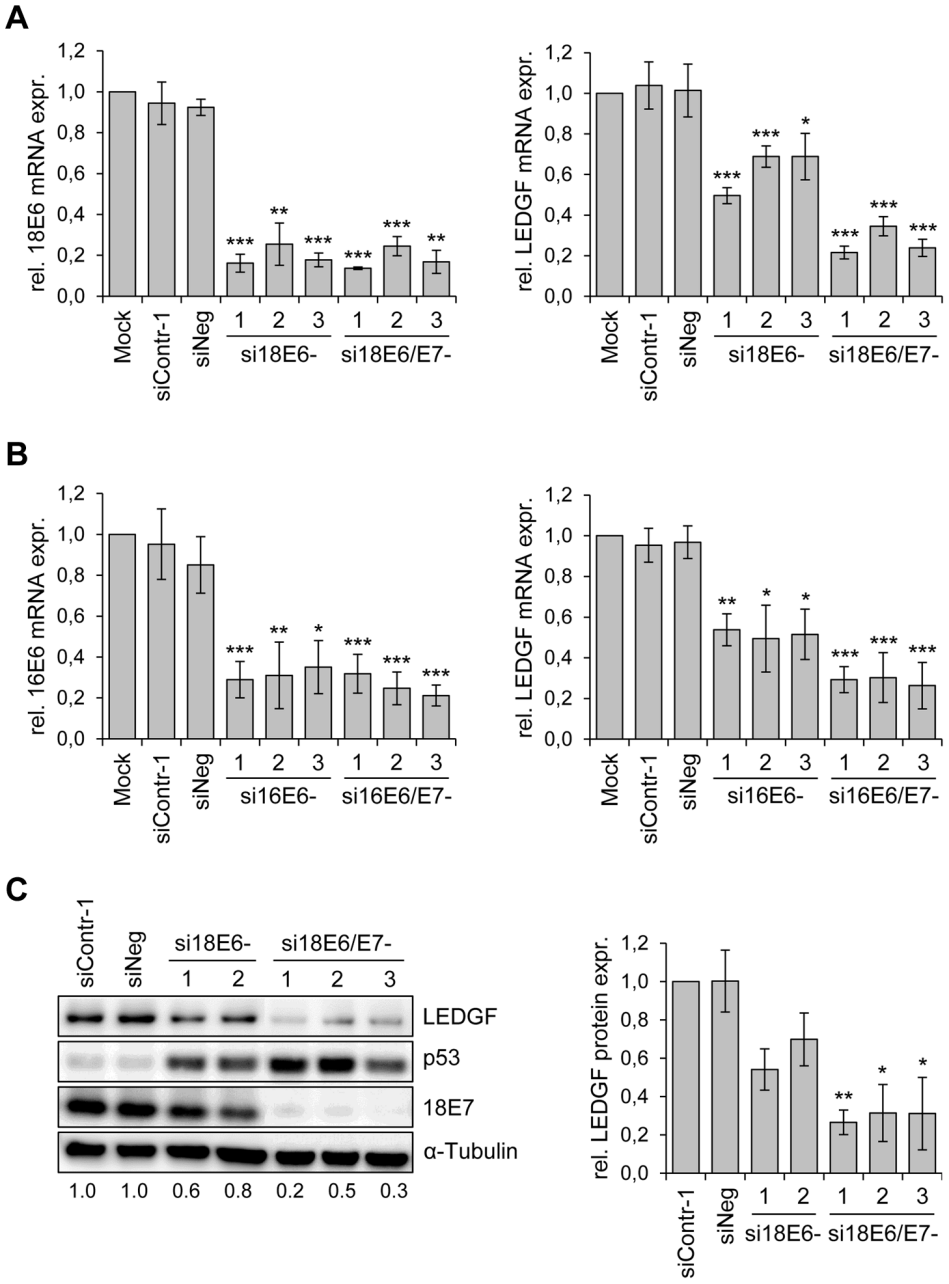
HPV oncogene silencing represses *LEDGF* expression. (**A**) siRNA-mediated silencing of HPV18 *E6* and *E6/E7* expression (left panel) and *LEDGF* transcript levels (right panel) in HPV18-positive HeLa cells. si18E6-1, -2, and -3: three different siRNAs specifically blocking HPV18 *E6* expression; si18E6/E7-1, -2, and -3: three different siRNAs concomitantly blocking HPV18 *E6* and *E7* expression. Mock: cells treated with transfection reagent only; siContr-1 and siNeg: control siRNAs. Indicated are relative mRNA levels determined by qRT-PCR, the value for mock-transfected cells was arbitrarily set at 1.0. Standard deviations are indicated. Asterisks above columns indicate statistically significant differences from mock-treated controls, with p-values of ≤0.05 (*),≤0.01 (**), or ≤0.001 (***). (**B**) siRNA-mediated silencing of HPV16 *E6* and *E6/E7* expression (left panel) and *LEDGF* transcript levels (right panel) in HPV16-positive SiHa cells. si16E6-1, -2, and -3: three different siRNAs specifically blocking HPV16 *E6* expression; si16E6/E7-1, -2, and -3: three different siRNAs concomitantly blocking HPV16 *E6* and *E7* expression. Asterisks above columns indicate statistically significant differences from mock-treated controls, with p-values of ≤0.05 (*),≤0.01 (**), or ≤0.001 (***). (**C**) Determination of LEDGF protein amounts. Left panel: Representative immunoblot analysis of HeLa cells. Shown are LEDGF, p53 and HPV18 E7 protein levels upon silencing of endogenous HPV18 *E6* (si18E6-1, and -2) or HPV18 *E6/E7* (si18E6/E7-1, -2, and -3) expression. α-Tubulin: loading control. Relative quantifications of LEDGF signal intensities are indicated below each lane, the value for siContr-1 transfected cells was set at 1.0. Right panel: Quantification of LEDGF protein levels in HeLa cells, densitometrically determined from three independent immunoblot analyses. Standard deviations are indicated. Asterisks above columns indicate statistically significant differences from siContr-1-transfected cells, with p-values of ≤0.05 (*) or ≤0.01 (**).

Previous data from a genomewide transcriptome array in HeLa cells indicated that *LEDGF* transcript levels are significantly reduced upon silencing of endogenous HPV18 *E6/E7* expression [13]. To confirm this result by independent methods, we tested the effects of HPV oncogene silencing on *LEDGF* expression by both qRT-PCR and immunoblot. We employed different siRNAs that either selectively block HPV *E6* expression or concomitantly block *E6* and *E7* expression from the polycistronic *E6/E7* transcripts [11]. As shown in Fig. 1A, these siRNAs efficiently reduced HPV18 mRNA amounts in HeLa cells. Inhibition of viral oncogene expression in HeLa cells was linked to a substantial reduction of *LEDGF* transcript levels upon combined *E6/E7* silencing whereas *E6* silencing alone inhibited *LEDGF* expression less strongly (Fig. 1A). *LEDGF* repression upon silencing of HPV *E6/E7* expression was neither specific for HPV18 nor a peculiarity of HeLa cells, since inhibition of endogenous *E6/E7* expression in HPV16-positive SiHa cells led to corresponding results as those observed in HPV18-positive HeLa cells (Fig. 1B).

In order to investigate whether the *E6/E7*-dependent *LEDGF* mRNA modulation translates into alterations of LEDGF protein levels, we performed Western blot analyses of siRNA-treated cells. In agreement with the E6 property to induce degradation of p53 [35], treatment of HeLa cells with siRNAs blocking *E6* or *E6/E7* expression led to an increase of p53 protein levels, and siRNAs blocking *E6/E7* expression additionally reduced E7 protein levels (Fig. 1C). Corresponding to the mRNA data, *E6/E7* silencing substantially reduced LEDGF protein levels whereas inhibition of *E6* expression alone reduced it less strongly. Taken together, these results show that continuous viral *E6/E7* oncogene expression is a crucial determinant for the maintenance of *LEDGF* expression in HPV-positive cancer cells.

### Activation of LEDGF expression by the HPV E6/E7 oncogenes

**Figure 2:**
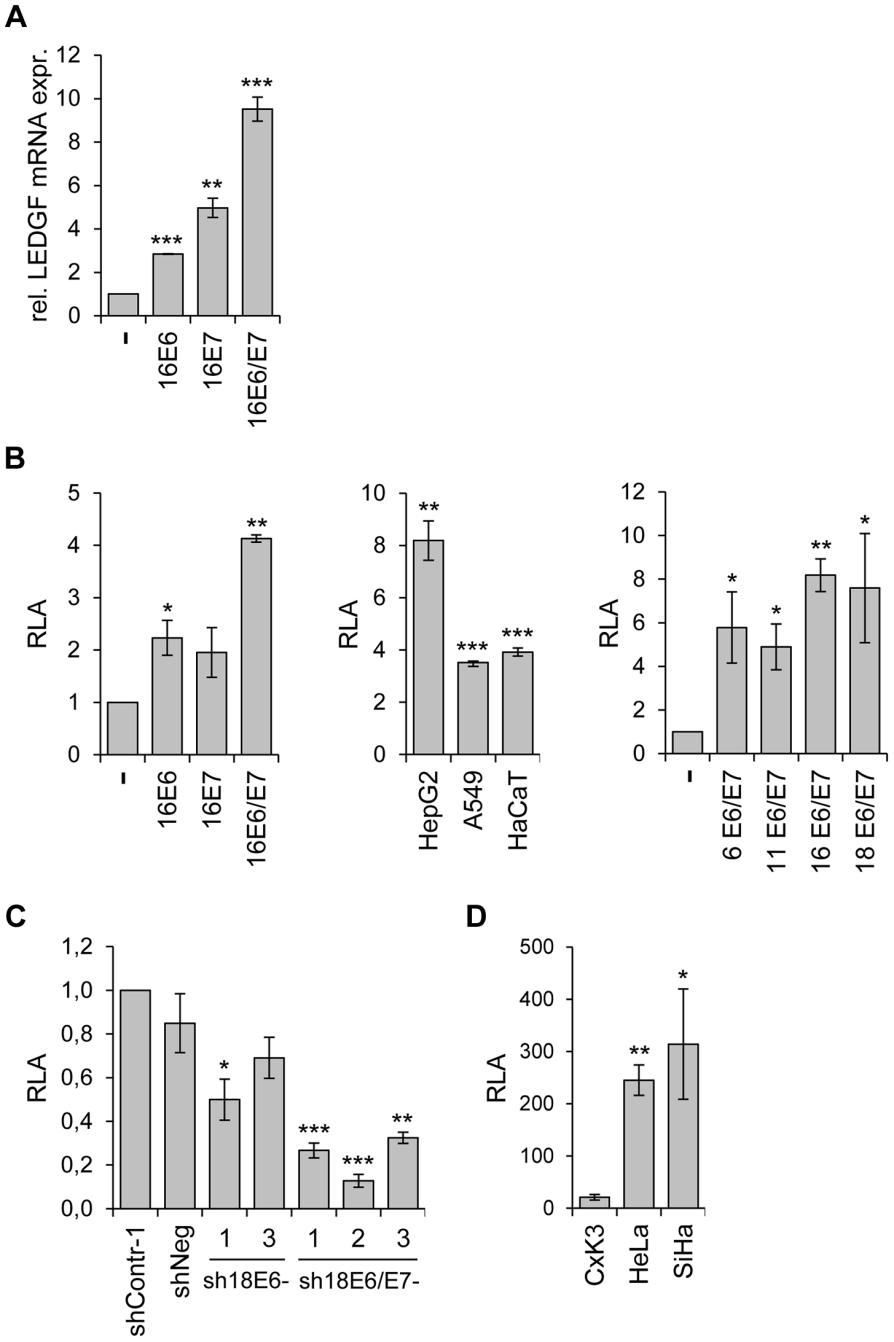
Activation of *LEDGF* expression by the HPV *E6/E7* oncogenes. (**A**) *LEDGF* transcript measurements by qRT-PCR. Primary human keratinocytes were transduced with retroviral vectors coding for HPV16 E6, -E7, or -E6/E7 and relative *LEDGF* transcript levels were determined (the value for keratinocytes transduced with the empty retroviral vector (-) was arbitrarily set at 1.0). Standard deviations are indicated. Asterisks above columns indicate statistically significant differences from control cells transduced with the empty retroviral vector, with p-values of ≤0.01 (**) or ≤0.001 (***). (**B**) Analysis of *LEDGF* promoter activities by luciferase reporter assay. Left panel: Primary keratinocytes were transfected with expression vectors coding for HPV16 E6, -E7, or -E6 and E7, together with a luciferase reporter plasmid (pGL4.10LEDGFp75 −723/+59) under transcriptional control of the *LEDGF* promoter [60]. Relative luciferase activities (RLA) are indicated above those of cells transfected with the empty expression vector (-), arbitrarily set at 1.0. Central panel: HepG2, A549 and HaCaT cells were transfected with expression vectors coding for HPV16 E6 and E7, together with pGL4.10LEDGFp75 −723/+59. RLA are indicated above those of respective control cells transfected with the empty expression vector (arbitrarily set at 1.0). Right panel: HepG2 cells were transfected with expression vectors coding for E6 and E7 from HPV6, HPV11, HPV16 and HPV18, together with pGL4.10LEDGFp75 −723/+59. Relative luciferase activities (RLA) are indicated above those of cells transfected with the empty expression vector (-), arbitrarily set at 1.0. Standard deviations are indicated. Asterisks above columns indicate statistically significant differences from cells transfected with the empty expression vector, with p-values of ≤0.05 (*),≤0.01 (**), or ≤0.001 (***). (**C**) Analysis of *LEDGF* promoter activities by luciferase reporter assay. HeLa cells were transfected with shRNA-expressing vectors blocking E6 (sh18E6-1, and -3) or E6/E7 (sh18E6/E7-1, -2, and -3) together with reporter plasmid pGL4.10LEDGFp75 −723/+59. shContr-1 and shNeg: negative controls. Luciferase activities are indicated relative to those for shContr-1-transfected cells (arbitrarily set at 1.0). Standard deviations are indicated. Asterisks above columns indicate statistically significant differences from shContr-1-transfected cells, with p-values of ≤0.05 (*),≤0.01 (**), or ≤0.001 (***). (**D**) Basal *LEDGF* promoter activities. Primary cervical keratinocytes (CxK3), HPV18-positive HeLa and HPV16-positive SiHa cervical carcinoma cells were transfected with reporter plasmid pGL4.10LEDGFp75 −723/+59. Values are indicated relative to corresponding control cells transfected with basic luciferase vector pGL4.10 (devoid of the *LEDGF* promoter fragment), arbitrarily set at 1.0. Standard deviations are indicated. Asterisks above columns indicate statistically significant differences from CxK3 cells, with p-values of ≤0.05 (*) or ≤0.01 (**).

The strong *LEDGF* repression observed upon *E6/E7* silencing raises the possibility that the viral oncogenes can activate *LEDGF* expression. To test this issue, we transduced primary human keratinocytes with retroviral vectors coding for HPV16 E6, E7 or E6/E7. Compared to control-transduced keratinocytes, both E6 and E7 alone activated endogenous *LEDGF* expression and the effect was enhanced when both viral genes were co-expressed (Fig. 2A). Activation of *LEDGF* expression by the HPV oncogenes occurred, at least in part, at the transcriptional level, as indicated by Luciferase-reporter assays. Both E6 and E7 alone weakly activated the *LEDGF* promoter upon ectopic expression in primary human keratinocytes and the stimulatory effect was enhanced when both viral oncogenes were co-expressed (Fig. 2B). Activation of the LEDGF promoter by co-expressed HPV16 E6 and E7 was not limited to primary keratinocytes but was also detectable in different tested epithelial cell lines (Fig. 2B). Moreover, the potential of E6/E7 expression to significantly activate the LEDGF promoter was not restricted to HPV16, but also observed for high risk HPV18 and for low risk HPV6 or HPV11 (Fig. 2B).

*Vice versa*, inhibition of endogenous *E6* or *E6/E7* expression by RNAi reduced *LEDGF* promoter activity in HeLa cells, with a stronger repression observed upon combined *E6/E7* silencing (Fig. 2C). In line with the notion that the enhancement of *LEDGF* gene expression in HPV-positive cancer cells occurs, at least in part, at the transcriptional level, HeLa and SiHa cells exhibit substantially higher basal *LEDGF* promoter activities than primary cervical keratinocytes (Fig. 2D).

**Figure 3:**
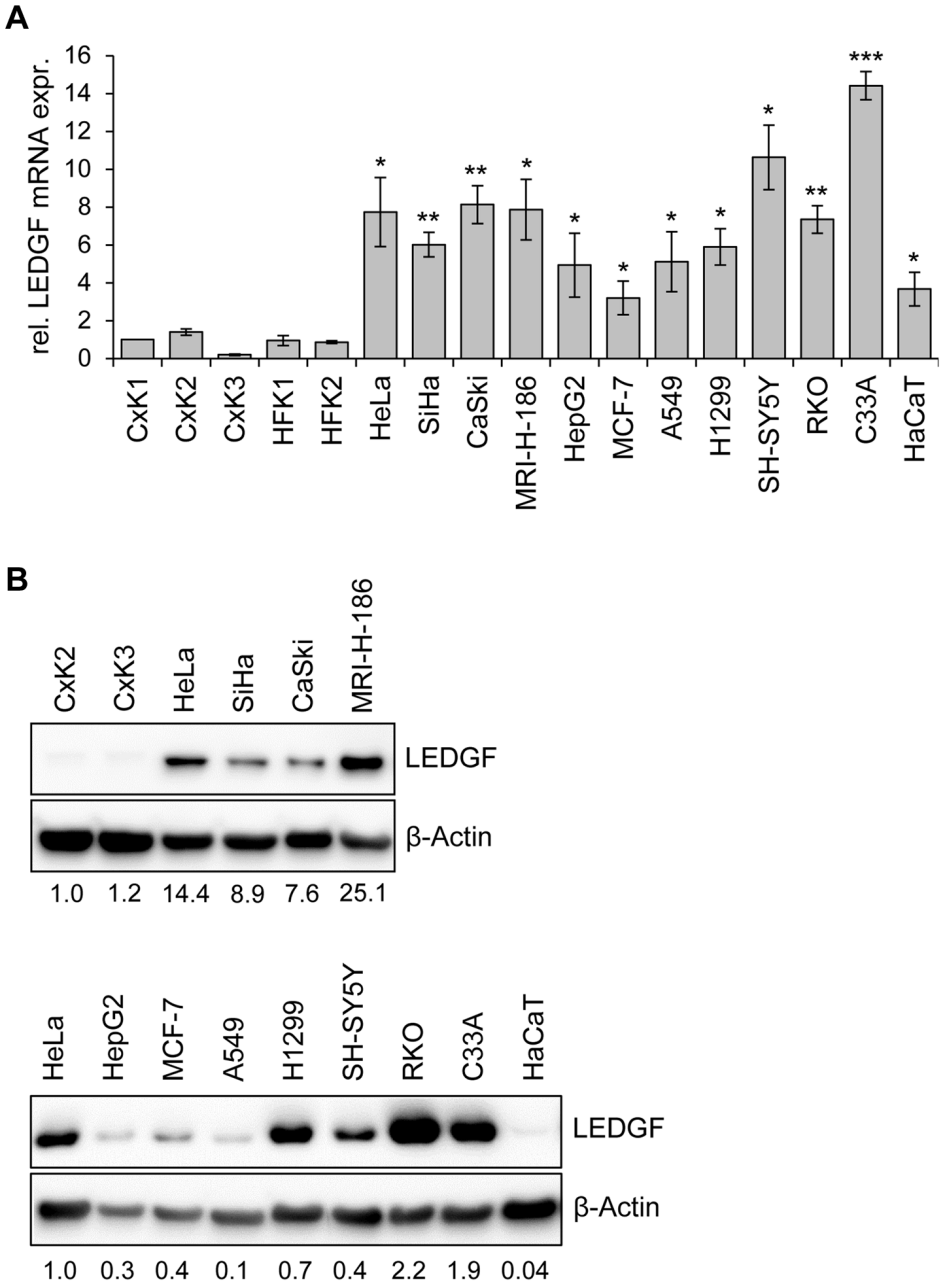
LEDGF expression in primary human keratinocytes and in HPV-positive and HPV-negative cell lines. (**A**) qRT-PCR analyses of basal *LEDGF* mRNA levels in primary human keratinocytes derived from the cervix uteri (CxK1, -2, and -3) or from foreskin (HFK1, and -2), and from a series of HPV-positive (HeLa, SiHa, CaSki, MRI-H-186) or HPV-negative (HepG2, MCF-7, A549, H1299, SH-SY5Y, RKO, C33A, HaCaT) cells. Values correspond to relative *LEDGF* transcript levels above those in cervical keratinocyte isolate CxK1 (arbitrarily set at 1.0). Standard deviations are indicated. Asterisks above columns indicate statistically significant differences between individual cell lines and CxK1, with p-values of ≤0.05 (*),≤0.01 (**), or ≤0.001 (***). (**B**) Representative immunoblot analysis of basal LEDGF protein expression. Upper panel: Comparison of HPV-positive cell lines (HeLa, SiHa, CaSki, MRI-H-186) with primary cervical keratinocyte isolates CxK2 and CxK3. Relative quantifications of LEDGF signal intensities are indicated below each lane, the value for CxK2 was set at 1.0. Lower panel: Comparison of LEDGF protein levels in HeLa cells with a series of HPV-negative cell lines. Relative quantifications of LEDGF signal intensities are indicated below each lane, the value for HeLa was set at 1.0. β-Actin: loading control.

If HPVs activate endogenous *LEDGF* expression, one would expect higher levels of LEDGF in HPV-positive cancer cells than in human keratinocytes, the natural target cells for HPV infection. To investigate this issue, we measured basal *LEDGF* mRNA and LEDGF protein levels in different isolates of primary human keratinocytes (from different donors), in a series of HPV16- and HPV18-positive cervical cancer cell lines, and in HPV-negative cell lines. Compared to primary foreskin or cervical keratinocytes, HPV18-positive HeLa and HPV16-positive SiHa, CaSki, and MRI-H-186 cells all exhibited elevated *LEDGF* expression levels, both at the transcript and protein level (Fig. 3). This increase in *LEDGF* mRNA and protein expression was not limited to HPV-positive cells and was quantitatively within the range of *LEDGF* expression levels in other, HPV-negative cell lines, e. g. lower than in C33A and higher than in HepG2 or MCF-7 (Fig. 3).

Taken together, these results show that *LEDGF* expression levels in HPV-positive cancer cells, as well as in other cancer cells, are higher than in primary keratinocytes. These observations are in line with the *LEDGF* upregulation reported for cancer biopsies from several different tumor types [18]–[20]. Importantly, in HPV-positive cancer cells, *LEDGF* expression is critically dependent on the maintenance of viral *E6/E7* oncogene expression.

### LEDGF expression in HPV-positive cancer cells is not altered by cell cycle arrest

**Figure 4:**
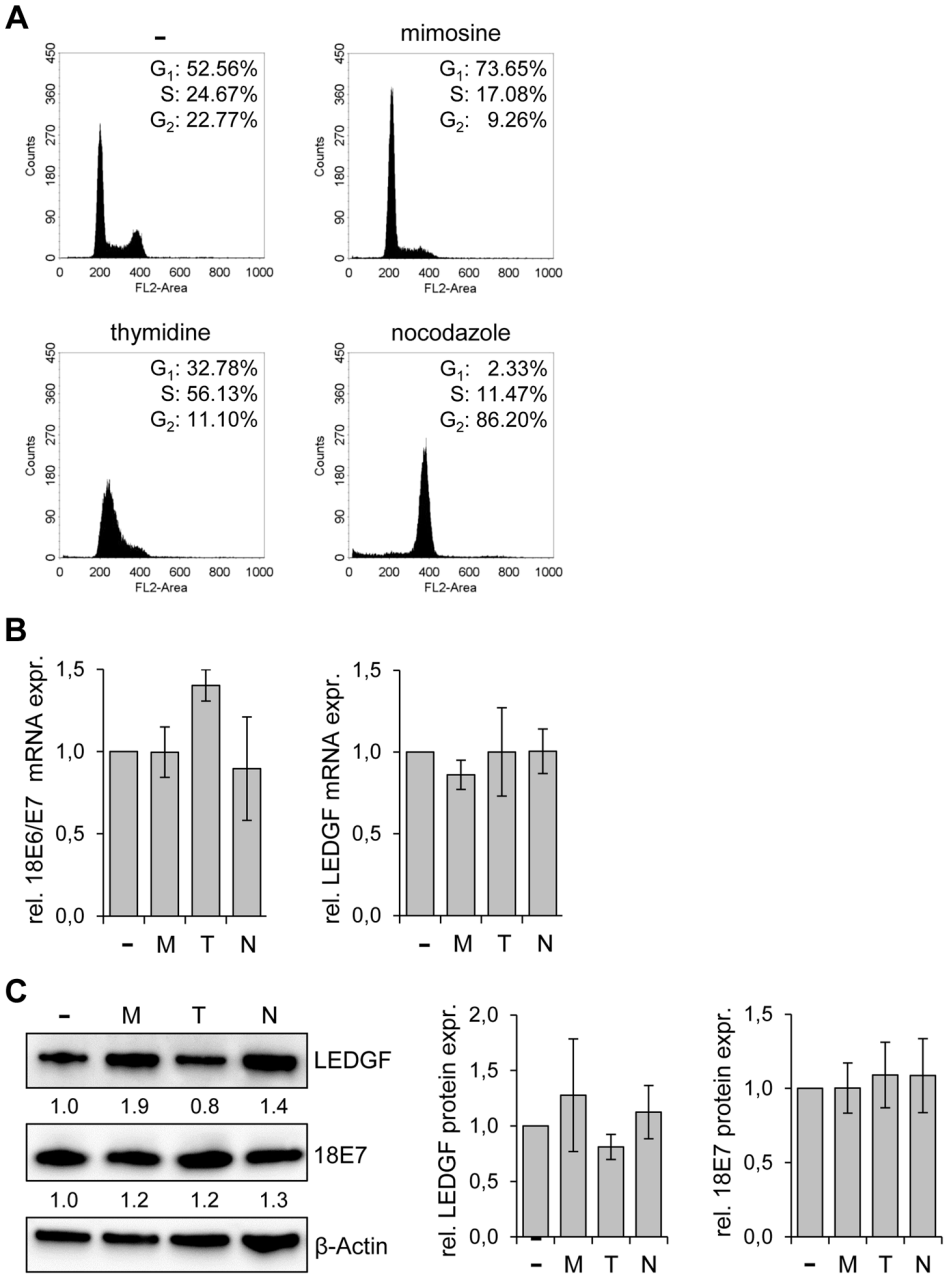
*LEDGF* expression in HeLa is not altered by cell cycle-inhibitory drugs. (**A**) Cell cycle distribution analyzed by FACS. HeLa cells were either left untreated (-) or treated with mimosine, thymidine or nocodazole. The percentages of cells in the G1, S, and G2 phases are indicated. (**B**) qRT-PCR analyses of *E6/E7* (left panel) and *LEDGF* (right panel) transcript levels in untreated cells (-) and in cells treated with either mimosine (M), thymidine (T) or nocodazole (N). Indicated are relative mRNA levels above those of untreated cells, arbitrarily set at 1.0. Standard deviations are indicated. Statistical analyses did not reveal significant differences between untreated and treated cells. (**C**) Analysis of LEDGF and HPV18 E7 protein levels upon treatment of HeLa cells with either mimosine (M), thymidine (T) or nocodazole (N). (-): untreated cells. β-Actin: loading control. Statistical analyses did not reveal significant differences between untreated and treated cells. Left panel: Representative immunoblot. Relative quantifications of LEDGF and HPV18 E7 signal intensities are indicated below the respective lanes, the value for untreated cells was set at 1.0. Right panel: Statistical analyses from three different immunoblots did not reveal significant differences between untreated and treated cells.

It is known that combined *E6/E7* silencing, either by the viral E2 transcriptional repressor [36], [37] or by RNAi [38], blocks proliferation of HPV-positive cancer cells by inducing a G1 cell cycle arrest. This raises the question whether the strong reduction of *LEDGF* expression upon *E6/E7* inhibition might be generally linked to an inhibition of cell cycle progression. In order to test this issue, we treated HeLa cells with chemical compounds that induce blocks in different cell cycle phases. Mimosine, thymidine, and nocodazole arrested the cells in the G1-phase, S-phase, and G2 phase, respectively (Fig. 4A), as expected for these drugs [39], [40]. However, none of the compounds significantly reduced *LEDGF* expression, neither at the transcript nor at the protein level (Figs. 4B and 4C). HPV *E6/E7* mRNA and E7 protein expression levels were also not significantly changed by the compounds (Figs. 4B and 4C). Thus, *LEDGF* expression was not decreased by different cell cycle inhibitory drugs, indicating that the reduction of *LEDGF* expression is not a secondary effect of the cell cycle arrest induced by *E6/E7* silencing.

### LEDGF silencing blocks the colony formation capacity of tumor cells in the presence of genotoxic agents

**Figure 5:**
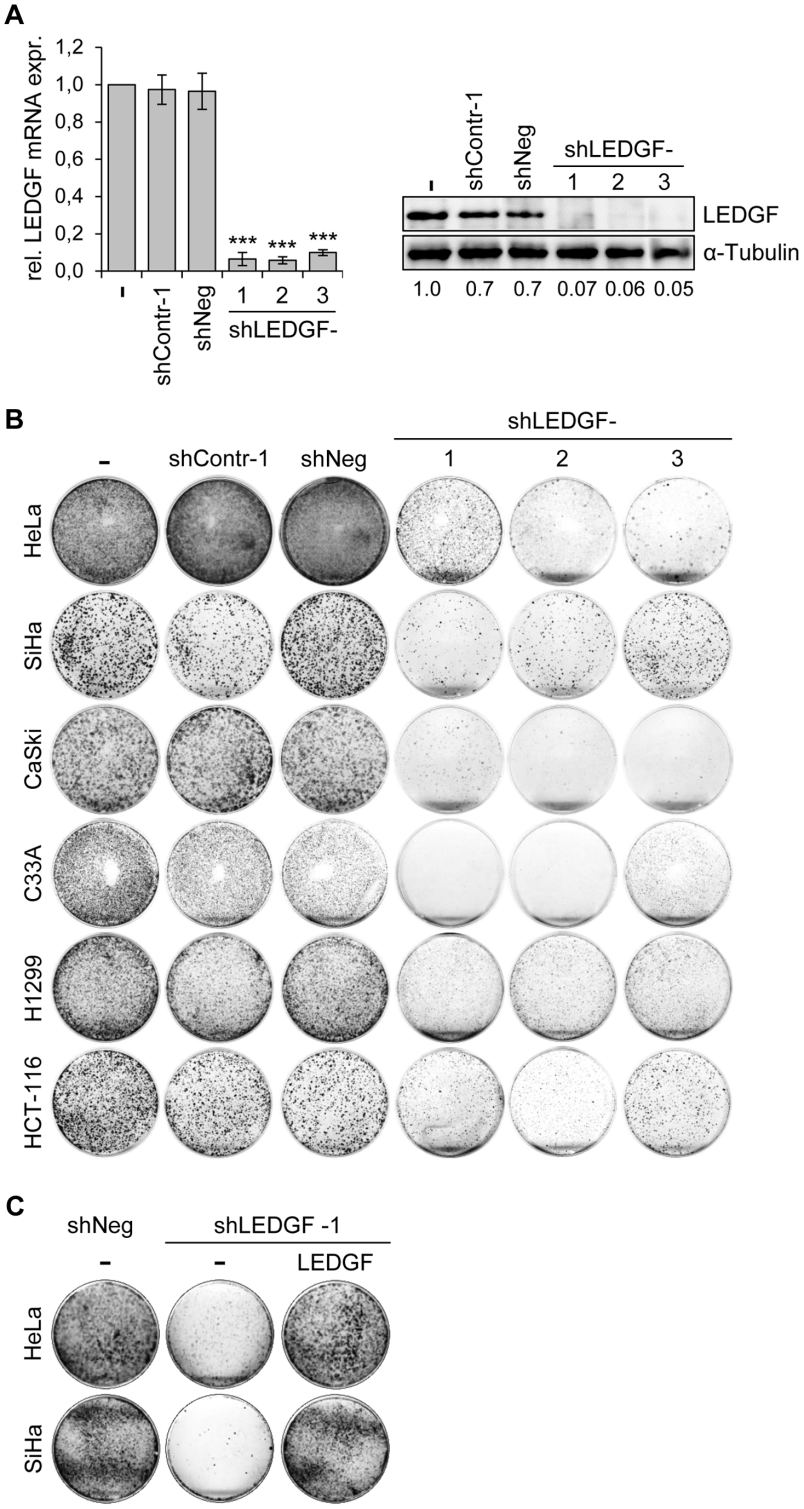
*LEDGF* silencing by shRNAs blocks the growth of tumor cell lines in colony formation assays (CFAs). (**A**) Inhibition of endogenous *LEDGF* expression by shRNAs. Left panel: HeLa cells were transfected with expression vectors for three different shRNAs blocking *LEDGF* expression (shLEDGF-1, -2, and -3) and *LEDGF* mRNA levels were determined by qRT-PCR. shContr-1 and shNeg: negative control shRNAs. (-): empty vector-transfected HeLa cells (set at 1.0). Standard deviations are indicated. Asterisks above columns indicate statistically significant differences from empty vector-transfected cells (set at 1.0), with p-values of ≤0.001 (***). Right panel: Corresponding immunoblot analysis of LEDGF protein expression. Densitometrically determined LEDGF signal intensities are shown below the lanes and are indicated relative to empty vector-transfected cells (-), set at 1.0. α-Tubulin: loading control. (**B**) CFAs of tumor cell lines upon stable transfection and hygromycin B selection for the shRNA-expressing plasmids characterized in (A). Cells were selected for 10–13 days, colonies were stained with crystal violet. (**C**) LEDGF reconstitution experiments in HPV-positive cells. CFAs of HeLa and SiHa cells upon stable transfection and hygromycin B selection for vectors expressing either negative control shNeg or shLEDGF-1, as indicated. Cells were concomitantly transfected with either a vector expressing wildtype LEDGF protein from a shLEDGF-1-resistent cDNA (LEDGF) or with the basic expression vector devoid of *LEDGF* sequences (-).

Next, we tested the phenotypic consequences of *LEDGF* modulation in HPV-positive cancer cells. We silenced endogenous *LEDGF* expression by stable transfection of plasmids expressing short-hairpin (sh)RNAs and performed colony formation assays (CFAs). For the shRNAs, we chose three different target sequences within the *LEDGF* mRNA, one of them (targeted by shLEDGF-3) being also present in the mRNA coding for the alternatively spliced LEDGF/p52 isoform [41]. All three shRNAs efficiently blocked *LEDGF* expression at the RNA and protein level (Fig. 5A). Compared to empty vector-transfected cells or cells transfected with vectors expressing control shRNAs (shContr-1, shNeg), HPV18-positive (HeLa) and HPV16-positive (SiHa, CaSki) cell lines all showed strongly reduced colony formation capacities upon silencing of endogenous *LEDGF* expression by each of the three different shRNAs (shLEDGF-1, -2, -3) (Fig. 5B). This effect was not limited to HPV-positive cells and was not linked to the p53 mutational status since *LEDGF* repression also resulted in a reduction of the colony formation capacity in HPV-negative C33A cervical carcinoma (mutant p53), H1299 lung cancer (p53 null) and HCT-116 colon carcinoma (wildtype p53) cells (Fig. 5B).

To corroborate that the reduction in colony numbers of HPV-positive cells was specifically due to *LEDGF* gene silencing, we performed LEDGF reconstitution experiments. We ectopically expressed the wildtype LEDGF protein from a cDNA in which we introduced silent mutations that confer resistance to the employed *LEDGF*-targeting shRNA (shLEDGF-1). This cDNA efficiently rescued the capacity of HeLa and SiHa cells to form colonies (Fig. 5C), confirming that the strong inhibitory effect of the *LEDGF*-targeting shRNAs on the growth of HPV-positive cell lines is due to the silencing of endogenous *LEDGF* expression.

**Figure 6:**
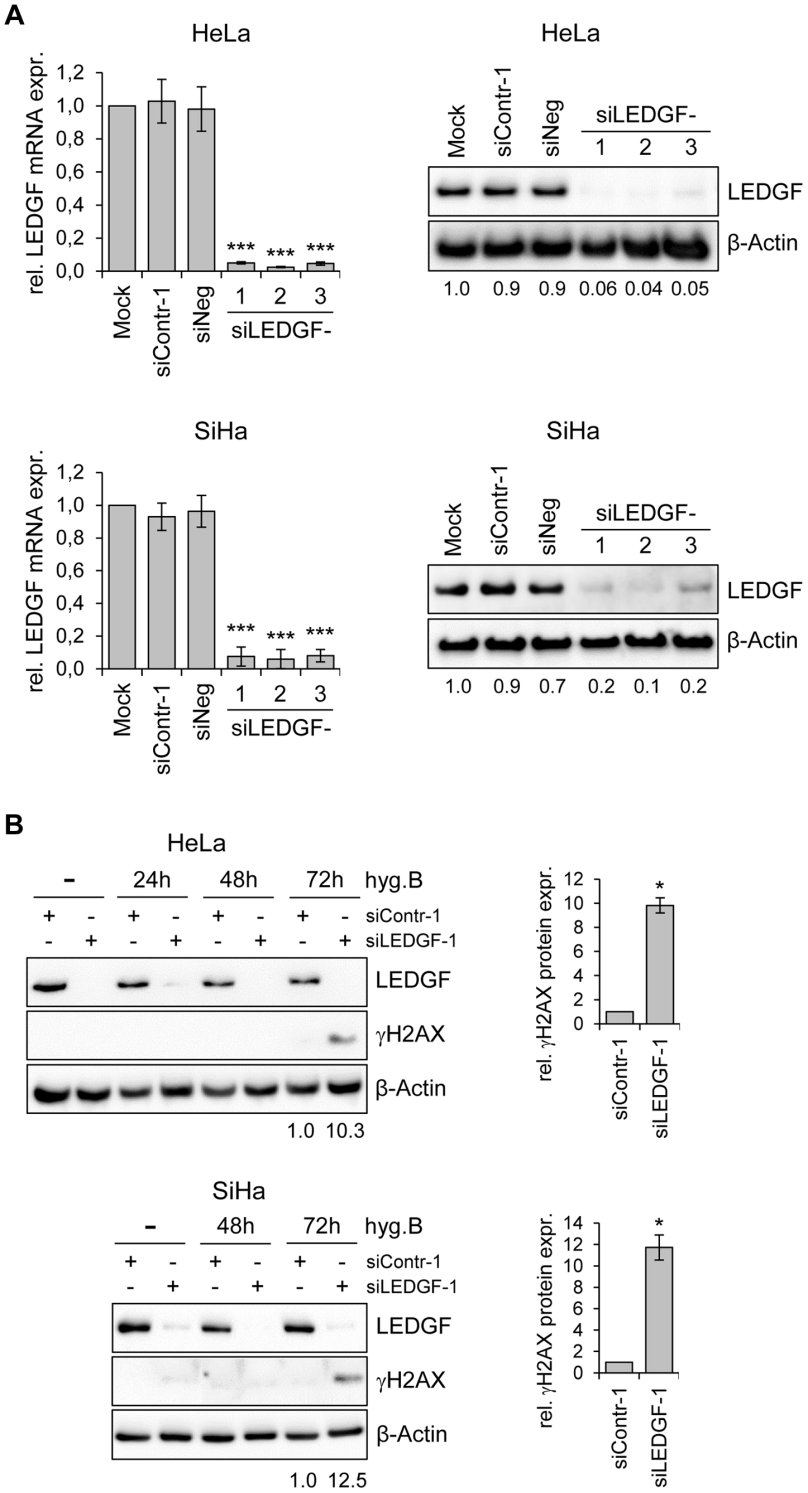
Silencing of *LEDGF* expression by synthetic siRNAs increases hygromycin B-induced genotoxicity in HPV-positive cancer cells. (**A**) RNAi-mediated inhibition of *LEDGF* mRNA and protein expression in HeLa (upper panels) or SiHa cells (lower panels). Cells were transfected with three different *LEDGF*-targeting siRNAs (siLEDGF-1, -2, and -3) or control siRNAs (siContr-1, siNeg). Left panels: Indicated are relative *LEDGF* mRNA concentrations (compared to mock-treated controls, arbitrarily set at 1.0), as determined by qRT-PCR. Standard deviations are indicated. Asterisks above columns indicate statistically significant differences from mock-treated control cells (set at 1.0) with p-values of ≤0.001 (***). Right panels: Representative immunoblots. Densitometrically determined LEDGF signal intensities are shown below each lane and are indicated relative to mock-treated control cells (arbitrarily set at 1.0). β-Actin: loading control. (**B**) Expression of LEDGF and the DNA damage marker γH2AX. Left panels: Immunoblot analyses of HeLa and SiHa cells, either transfected with siLEDGF-1 or control siContr-1, and treated with 200 µg/ml hygromycin B (hygB) for the indicated time periods. Densitometrically determined γH2AX signal intensities in LEDGF-depleted cells (siLEDGF-1) at 72 h are shown below the respective lanes and are indicated relative to the values of siContr-1-transfected cells at 72 h (arbitrarily set at 1.0). β-Actin: loading control. Right panels: Quantification of γH2AX signal intensities at 72 h from two independent experiments. Asterisks above columns indicate statistically significant differences from siContr-1-transfected cells (set at 1.0), with p-values of ≤0.05 (*).

These findings indicate that *LEDGF* silencing substantially inhibits the growth of HPV-positive cancer cells, as well as of other cancer cells, in CFAs. In order to get more insight into the underlying mechanism, we transiently transfected HeLa cells with synthetic siRNAs targeting *LEDGF* and tested possible effects of LEDGF depletion on cellular growth or apoptosis control. Surprisingly, and in apparent discrepancy to the prominent effects seen in CFAs (Fig. 5), we observed only a relatively modest influence on cell growth, cell cycle distribution, or apoptosis rate (data not shown), although the transiently transfected siRNAs led to efficient silencing of endogenous *LEDGF* expression, both at the RNA and protein level (Fig. 6A).

An experimental difference between the stable and transient transfection studies performed here is the presence of hygromycin B in the cell culture medium for the former, in order to select for the maintenance of the shRNA-expressing plasmid vectors. Hygromycin B is an aminoglycoside antibiotic that is classically known for its inhibitory activity on protein biosynthesis [42]. However, hygromycin B has also been reported to possess DNA-damaging potential [43]. Therefore, we treated HeLa and SiHa cells with hygromycin B and modulated endogenous *LEDGF* expression by siRNAs. Interestingly, a significant induction of the DNA damage marker γH2AX (phosphorylated form of H2A histone family member X) [44] was observed when hygromycin B-treated HeLa or SiHa cells were depleted for LEDGF (Fig. 6B), indicating that *LEDGF* silencing increases the genotoxic potential of hygromycin B.

**Figure 7:**
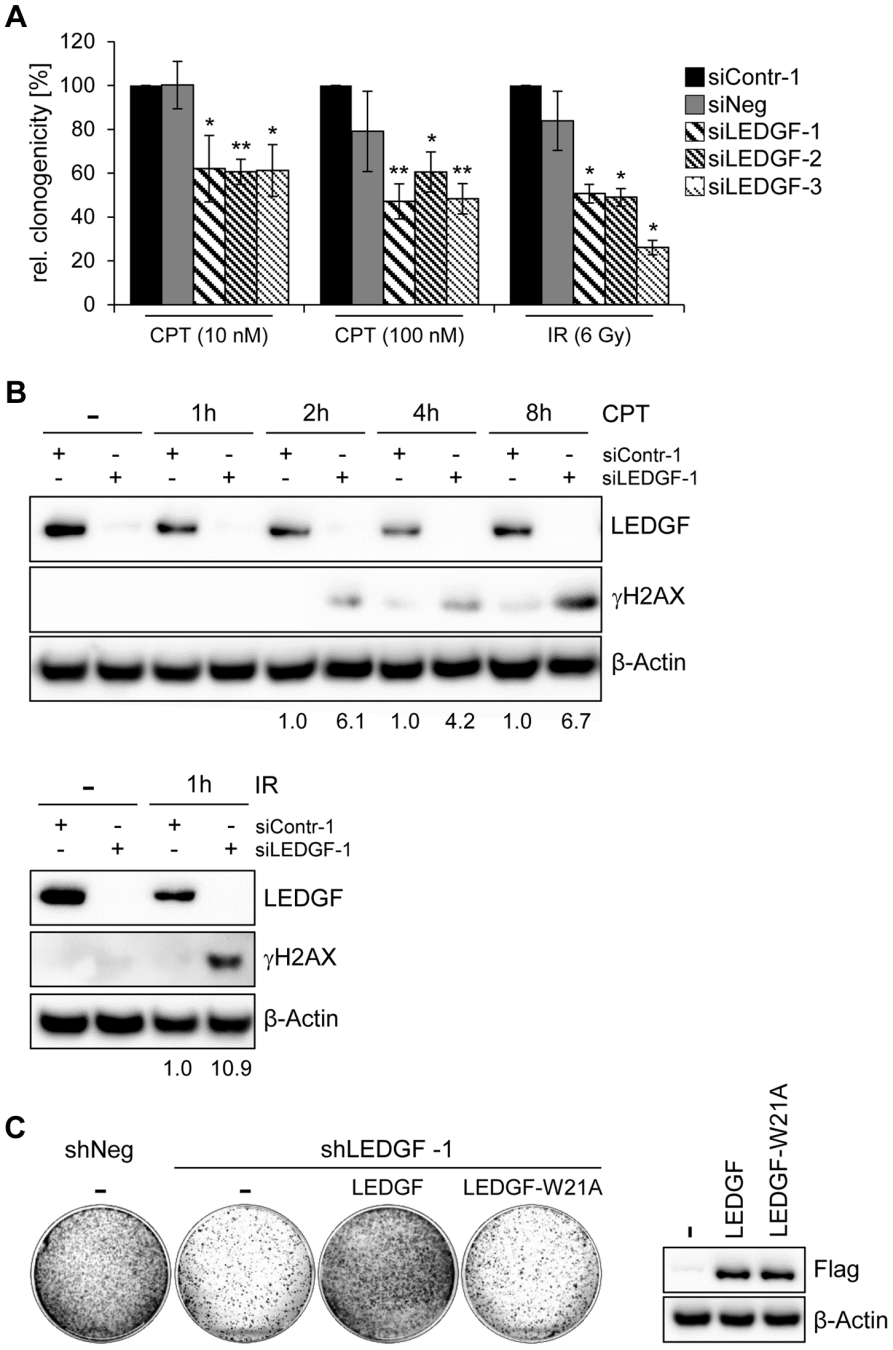
*LEDGF* silencing sensitizes HeLa cells towards genotoxic agents. (**A**) Cells were transfected with the synthetic siRNAs characterized in Fig. 6A and treated with different concentrations of camptothecin (CPT) or 6 Gy γ-irradiation, as indicated. Measured are relative clonogenicities, compared to siContr-1-transfected cells, in the presence of the respective drugs (arbitrarily set at 100%). Asterisks above columns indicate statistical significant differences, with p-values of ≤0.05 (*) and ≤0.01 (**). Standard deviations are indicated. (**B**) Immunoblot analyzing expression of LEDGF and of the DNA damage marker γH2AX. HeLa cells were transfected with the indicated synthetic siRNAs and treated with either 1 µM CPT for the indicated time periods (upper panel) or 6 Gy γ-irradiation (lower panel). Densitometrically determined γH2AX signal intensities in LEDGF-depleted cells (siLEDGF-1) are shown below the respective lanes and are indicated relative to the values of siContr-1-transfected cells, at the same time points (arbitrarily set at 1.0). β-Actin: loading control. (**C**) LEDGF reconstitution experiments. Left panel: CFAs of HeLa cells upon transfection and hygromycin B selection for vectors expressing control shRNA shNeg or the *LEDGF* mRNA-targeting shLEDGF-1. Cells were concomitantly transfected with either a vector expressing wildtype LEDGF protein (LEDGF) or LEDGF-W21A mutant protein (LEDGF-W21A) from shLEDGF-1-resistent cDNAs, or with the basic expression vector devoid of *LEDGF* sequences (-). Right panel: Wildtype LEDGF and LEDGF-W21A were expressed at comparable levels, as shown by an immunoblot employing a Flag-Tag antibody. β-Actin: loading control.

On the basis of these experiments, we hypothesized that the reduced colony formation capacity observed in stable transfection experiments (i. e. in the presence of hygromycin B) could be linked to a reduced protection of LEDGF-depleted cells against DNA damage. We therefore performed CFAs upon transient transfection with synthetic siRNAs and treatment with well-characterized DNA damaging agents. We found that *LEDGF* silencing in HeLa cells led to an increased sensitivity towards both the topoisomerase inhibitor camptothecin (CPT) and γ-irradiation, leading to significant reductions of colony formation capacities (Fig. 7A). This effect was linked to a strong γH2AX increase when cells were depleted for LEDGF (Fig. 7B). These data indicate that LEDGF plays an important role for protecting HPV-positive cells against DNA damage exerted by genotoxic drugs (CPT, hygromycin B) or γ-irradiation which is also supported by a recent study showing that LEDGF is involved in DNA repair [33]. In line, ectopic expression of a mutant LEDGF protein (LEDGF-W21A) which has lost its genoprotective activity [33] no longer could revert the inhibitory effect of endogenous LEDGF depletion on the colony formation capacity of HeLa cells, in the presence of hygromycin B (Fig. 7C). Taken together, these results indicate that the activation of *LEDGF* expression by the HPV *E6/E7* oncogenes plays an important role for the resistance of HPV-positive cancer cells towards genotoxic agents.

### LEDGF is significantly overexpressed in HPV-positive lesions in vivo

Finally, we tested whether the observed positive correlation between HPV *E6/E7* and *LEDGF* expression *in vitro* is also found *in vivo*. To this end, we analyzed LEDGF protein expression by immunohistochemistry in patient biopsies representing different degrees of premalignant cervical lesions (cervical intraepithelial neoplasia, CIN): CIN I (n = 16), CIN II (n = 7), CIN III (n = 13), and established squamous cell carcinomas (n = 7). The concomitant assessment of p16 protein expression served as a surrogate marker for HPV oncogene expression [45].

First, we tested the specificity of anti-LEDGF antibody (6E4) to be employed for LEDGF detection. Untreated HeLa cells, HeLa cells transfected with an siRNA silencing endogenous *LEDGF* expression, and HeLa cells in which LEDGF was ectopically overexpressed were prepared on thin-layer cytology slides. The cells were subsequently analyzed for LEDGF protein expression, employing our immunohistochemistry staining protocol. LEDGF protein was readily detectable in the nuclei of untreated HeLa cells (Supplemental Fig. S1). RNAi-mediated *LEDGF* gene silencing virtually completely extinguished the LEDGF signals whereas ectopic LEDGF overexpression resulted in augmented LEDGF signals when compared with untreated HeLa cells (Supplemental Fig. S1). These experiments indicate that the antibody is specific for LEDGF and suitable for LEDGF detection by immunohistochemistry.

**Figure 8:**
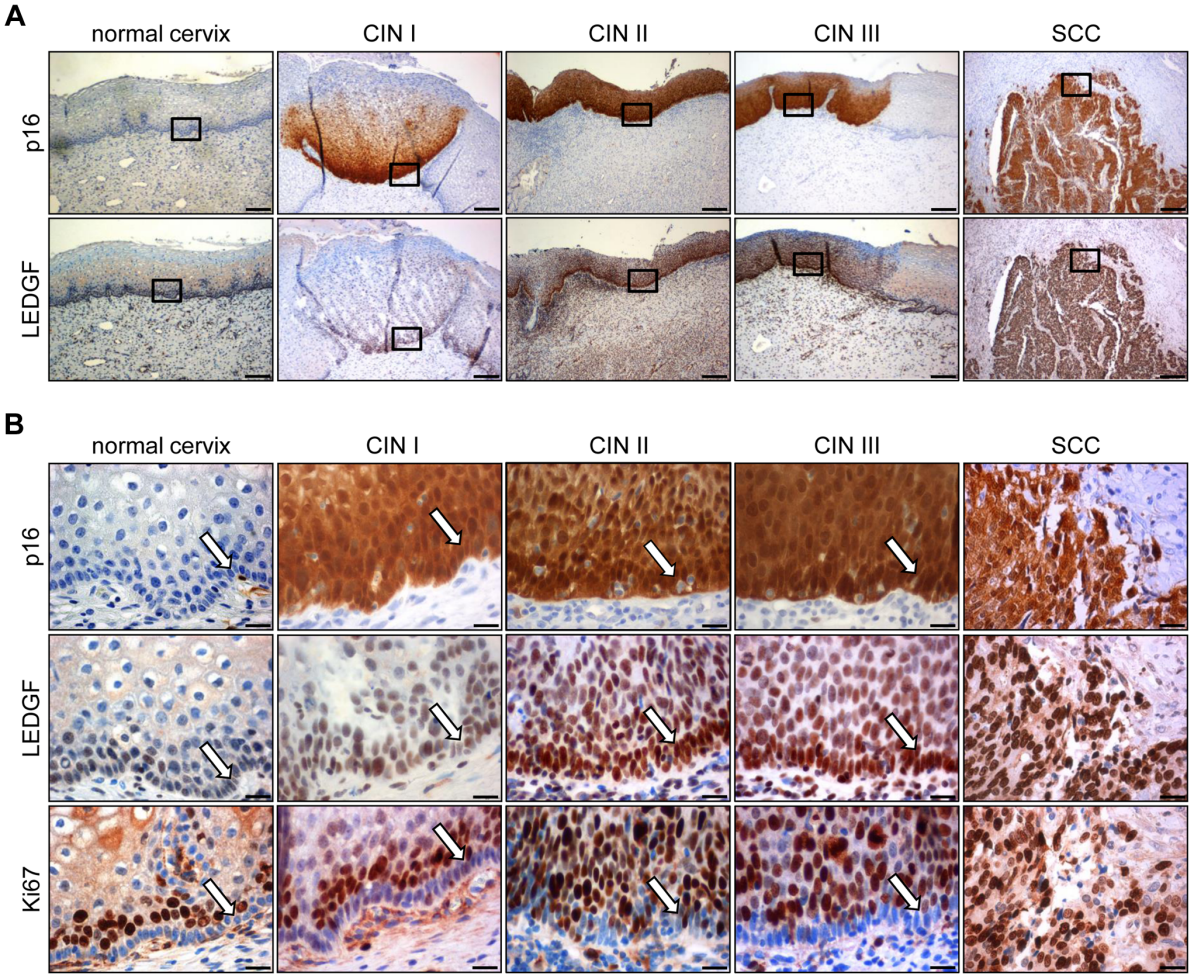
Immunohistochemical analysis of LEDGF expression. (**A**) Expression of LEDGF in histologically normal cervical epithelium, in dysplastic CIN I to CIN III lesions, and in cervical squamous cell carcinoma (SCC). p16: surrogate marker for HPV oncogene expression. Bars correspond to 200 µm. (**B**) Higher magnification of normal cervix, CIN I to III lesions, and cervical SCC. Staining of p16, LEDGF and proliferation marker Ki67. Arrows indicate the basal cell layer in normal cervix and CIN lesions. Bars correspond to 20 µm.

Analysis of histologically normal, p16-negative cervical epithelium revealed that LEDGF protein expression mainly localized to the basal and suprabasal cell layers (Fig. 8A and 8B). In comparison, epithelial LEDGF levels were clearly increased both in HPV-positive preneoplastic lesions and in established cervical cancers, overlapping with p16 signals in serial tissue sections (Fig. 8A). This finding is consistent with the positive correlation between HPV *E6/E7* and *LEDGF* expression found *in vitro*.

As observed for HeLa cells (Supplemental Fig. S1), LEDGF located primarily to the nuclei of the cells in the tissue sections (Fig. 8B). Notably, the strong LEDGF signals in the basal cell layers of both histologically normal and dysplastic HPV-positive cervical epithelium differed markedly from the expression of the Ki67 proliferation marker which was largely absent in the basal cell layer but was readily detectable in suprabasal cells (Fig. 8B).

**Figure 9:**
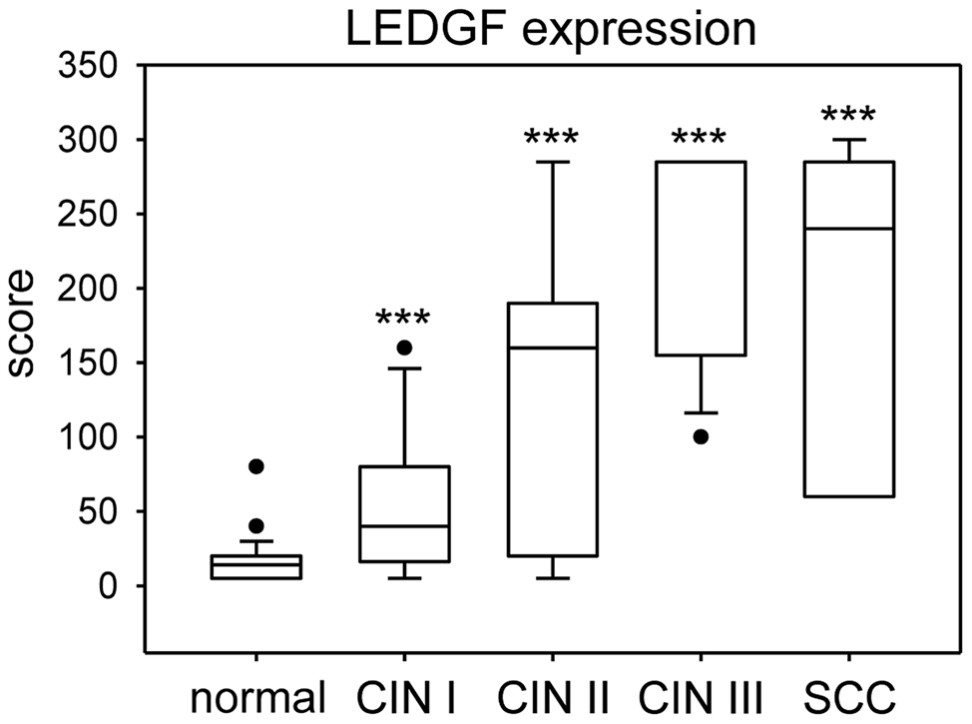
Box plot of LEDGF protein expression in cervical tissue. LEDGF expression was significantly increased in dysplastic lesions (CIN I: n = 16; CIN II: n = 7; CIN III: n = 13) and cervical cancer (n = 7) when compared with histologically normal, p16-negative cervical epithelium (n = 36). Asterisks above columns indicate statistically significant differences from histologically normal cervix, with p-values of ≤0.001 (***). Individual points in the graph represent outliers. Note that the median line for CIN III overlaps with the 75% quartile. The data is further specified in Supplemental Table S1.

In order to quantitatively assess LEDGF expression, we employed a score that considers both the percentage of cells positive for LEDGF as well as LEDGF staining intensity (Supplemental Table S1). Box plot analyses showed that epithelial LEDGF levels were statistically highly significant increased both in HPV-positive preneoplastic lesions and in established cervical cancers when compared with histologically normal, p16-negative epithelium (Fig. 9). In addition, there was a trend that LEDGF expression in cervical epithelium levels increases from mild to severe dysplasias to cancer (CIN I vs. CIN II, p = 0.021; CIN II vs. CIN III, p = 0.17; CIN III vs. cervical cancer p = 0.7). Taken together, these findings reveal a highly significant correlation between HPV-positivity and LEDGF expression levels *in vivo*, consistent with the *in vitro* data indicating activation and maintenance of LEDGF expression by the HPV oncogenes.

## Discussion

In this study, we identify the cellular *LEDGF* gene as a novel target for oncogenic HPVs. We show that continuous *E6/E7* oncogene expression is required to maintain intracellular *LEDGF* expression in HPV-positive cancer cells and that HPVs can transcriptionally stimulate the *LEDGF* gene via *LEDGF* promoter activation. Further, *LEDGF* expression is crucial for the resistance of HPV-positive cancer cells towards genotoxic stress. In line with the *in vitro* data demonstrating a positive correlation between HPV oncogene and *LEDGF* expression, we found that HPV-positive preneoplastic and neoplastic lesions exhibit significantly enhanced levels of LEDGF. We propose that stimulation of *LEDGF* expression by the viral *E6/E7* oncogenes is a crucial mechanism to protect HPV-positive cancer cells towards different forms of cellular stress, including DNA damage.

LEDGF is increasingly recognized as a factor involved in human tumorigenesis (see Introduction). Despite of the term “growth factor” in its name - which is based on its structural relatedness to hepatoma-derived growth factors [46] - it is currently uncertain whether LEDGF is secreted and serves as a classical growth factor [27], [47]. LEDGF possesses a nuclear localization signal [48] and is tightly bound to chromatin [49], [50]. The protein has been originally identified as a transcriptional coactivator interacting with components of the basal transcriptional machinery [41] and subsequently has been reported to stimulate expression of stress-related and cytoprotective genes, including the *heat shock protein HSP27* and the *antioxidant protein-2 (AOP2)* genes [51], [52].

LEDGF has been reported to undergo several protein-protein interactions that could be significant for tumorigenesis. For example, LEDGF acts as a chromatin tether for a trimeric complex with Menin and the MLL (mixed-lineage leukemia) histone methyltransferase which is essential for leukemic transformation by MLL oncoproteins [23]. In addition, LEDGF can bind to and tether the Myc-interacting protein JPO2 to chromatin [53], a factor that may possess transforming potential by augmenting the oncogenicity of c-Myc [54]. Recently, LEDGF has been found to associate with CtIP (C-terminal binding protein interacting protein) [33], a multifunctional adaptor protein with tumor suppressive potential [55]. Among other functions, such as cell cycle control [55], CtIP plays an important role for the repair of DNA double strand breaks (DSBs) by homologous recombination [56]. The important observation that LEDGF is critical for the access of CtIP to DNA DSBs [33] could provide a mechanistic explanation for the genoprotective activity of LEDGF.

This latter activity is also likely to account for the pronounced inhibition of LEDGF-depleted tumor cells in CFAs, in the presence of hygromycin B to select for stably transfected shRNA plasmids. In line with reports that aminoglycosides can induce single and double strand DNA breaks [43], [57], [58], we observed induction of the DNA damage marker γH2AX [44] when hygromycin B-treated HeLa and SiHa cells were depleted for LEDGF, although we cannot formally exclude that impurities in commercially available hygromycin B solutions may contribute to genotoxicity. In addition, inhibition of colony formation capacity in these HPV-positive tumor cells could be reverted by ectopic expression of wildtype LEDGF protein but not by a mutant LEDGF protein that has lost its genoprotective function. Taken together, our results indicate that the maintenance of *LEDGF* expression by the HPV oncogenes is an important determinant to allow growth of HPV-positive cells in the presence of genotoxic stress.

It is unlikely that *LEDGF* repression upon *E6/E7* inhibition is a secondary result of the accompanying cell cycle arrest, since *LEDGF* expression levels remained largely unchanged upon treatment of HPV-positive cervical cancer cells with different chemical compounds that block cell cycle progression. The notion that *LEDGF* expression levels are not simply proliferation-linked is further supported by the immunohistochemistry data. We found substantial LEDGF expression in the basal cell layers of both histologically normal and dysplastic cervical epithelium (Fig. 8C). This markedly differed from the expression of the Ki67 proliferation marker which was largely absent in the basal cell layer but was strongly expressed in the suprabasal layer, in line with the notion that suprabasal cells represent the main proliferative pool whereas basal cells contribute only little to the proliferative activity of the cervical epithelium [59]. Thus, the high levels of LEDGF protein in the basal cells that stain negative for Ki67 also suggest that pronounced *LEDGF* expression is not necessarily linked to a high proliferative index.

Several lines of experimental evidence indicate that HPVs can activate the *LEDGF* gene. Ectopic *E6/E7* expression in primary human keratinocytes, the natural target cells for HPVs, increased *LEDGF* mRNA levels. This was linked to enhanced activities of the *LEDGF* transcriptional promoter, as shown in reporter gene assays. *Vice versa*, silencing of endogenous *E6/E7* expression in HeLa cells repressed the *LEDGF* promoter but did not lead to alterations in the half-life of the *LEDGF* mRNA (data not shown). Expression of *E6* or *E7* alone less strongly stimulated *LEDGF* expression than the combined expression of both viral oncogenes, suggesting some degree of functional cooperativity during stimulation of *LEDGF* transcription. Unfortunately, the understanding of the transcriptional control of the *LEDGF* gene is still at an early stage. Somewhat discrepant results concerning the *LEDGF/p75* promoter have been reported by two research groups who mapped by transcriptional start site analyses a TATA-less promoter to two different genomic sites that are separated by 208 nucleotides [60], [61]. In our reporter gene assays, we employed a 782 bp fragment 5′ of the *LEDGF* gene which encompasses both putative promoters (fragment −723/+59; [60]).

Activation of the LEDGF promoter by E6/E7 was not limited to the oncogenic types HPV16 and HPV18, but also detectable for HPV6 and HPV11, two HPV types that are rarely associated with malignancy. It will be interesting to investigate a possible role for LEDGF in the viral life cycle of HPVs. Conceivably, HPVs may profit from upregulating stress-protective and pro-survival genes like LEDGF, thereby protecting the infected host cell during virus replication and synthesis.

Little is known about the specific transcriptional regulators involved in *LEDGF* promoter control, except for a stimulatory role found for the ubiquitous transcription factor SP1 [60], [61] and the observation that putative SP1 recognition sites within the *LEDGF* promoter can be targeted for epigenetic repression [62]. That *LEDGF* gene expression is indeed considerably regulated at the promoter level is also supported by the observation that basal *LEDGF* promoter activities were substantially enhanced in HPV-positive cancer cells above those in primary cervical keratinocytes, concomitantly with increased *LEDGF* mRNA and LEDGF protein levels in the former cells.

The exact intracellular localization of the LEDGF protein is still under some controversy. It has been described by some researchers to be predominantly nuclear [48], [63]–[65] whereas others additionally reported varying degrees of a cytoplasmic distribution [18], [20], [66], or a differentiation-dependent localization with nuclear LEDGF in the basal cell layer and cytoplasmic LEDGF in more differentiated cells of the epidermis [67]. The investigation of tissue sections of cervical epithelium revealed a predominantly nuclear LEDGF localization. Importantly, and consistent with the positive correlation between HPV oncogene and LEDGF expression levels observed *in vitro*, we found that p16-positive regions in patient biopsies exhibited statistically highly significant increased LEDGF expression levels when compared with p16-negative, histologically normal areas from the same tissue sections. In addition, there was a non-significant trend that LEDGF levels increased with increasing severity of dysplastic lesions to established cervical cancer. The latter finding is reminiscent of a study in bladder cancer, reporting a tendency for increasing LEDGF levels during tumor progression [19].

It is interesting that the basal cell layer - in both histologically normal cervical epithelium as well as in dysplastic lesions - exhibited prominent LEDGF staining. This cell layer also harbors the stem cells of the cervix [68]. Notably, a study in brain tissue reported LEDGF staining in neuroepithelial stem cells [66]. It is tempting to speculate that LEDGF may play a role for protecting stem cells, including stem cells of the cervical epithelium, against various forms of cellular stress. Stress factors that have been shown to be counteracted by LEDGF include serum starvation [28], [29], [31], oxidative stress [20], [27], [30], [51], [69], alcohol toxicity [32], thermal stress [27], [29], [69], and DNA damage [19], [22], [30], [33], [34].

In view of these multiple pro-survival activities of LEDGF, tumor cells should benefit from upregulating *LEDGF* expression. Indeed, the increased LEDGF levels in many different tumor entities, despite of their genetic heterogenicity, suggests a broadly relevant role for LEDGF in human carcinogenesis. The mechanisms of how tumor cells achieve upregulation of *LEDGF* expression are not understood. Our results provide the first evidence that the HPV oncogenes stimulate and maintain *LEDGF* expression in cervical cancer cells. It will be interesting for future studies to investigate whether the capacity to increase *LEDGF* expression is also shared by other viral and cellular oncogenes.

Under clinical aspects, the *E6/E7*-dependent maintenance of LEDGF expression could play a role for the therapeutic resistance of HPV-positive cancers, by protecting against the genotoxic effects of chemo- and radiotherapy. This raises the possibility that a combination of chemo- and/or radiotherapeutic agents with LEDGF inhibitors could increase the therapeutic sensitivity of cervical cancer cells and other tumor cells.

Finally, the *E6/E7*-dependent *LEDGF* expression may not only promote tumor growth by protecting HPV-positive cancer cells against different forms of cellular stress, but also could contribute to tumor progression and metastasis more directly, e.g. by enhancing the formation of blood and lymph vessels, as reported for the LEDGF-mediated activation of VEGF-C in glioma, lung cancer and ovarial cancer models [24], [26].

## Materials and Methods

### Cell culture, transfections and treatment conditions

HPV18-positive HeLa cervical carcinoma cells, HPV16-positive CaSki, MRI-H-186 and SiHa cervical carcinoma cells, HPV-negative C33A cervical carcinoma cells, HaCaT human immortal keratinocytes, H1299 and A549 lung cancer, RKO and HCT116 colon cancer, MCF-7 breast cancer, SH-SY5Y neuroblastoma and HepG2 hepatoma cells were maintained either in DMEM (pH 7.2), McCoy's, or RPMI medium (Gibco, Life Technologies, Carlsbad, CA), supplemented with 10% fetal bovine serum (FBS; Gibco Life Technologies), 2 mM L-glutamine, 100 U/ml penicillin and 100 µg/ml streptomycin (Sigma-Aldrich, Saint Lois, MO). Primary human cervical keratinocytes (CxK) or primary human foreskin keratinocytes (HFK) were grown in keratinocyte growth medium 2, supplemented with 0.06 mM CaCl_2_ and SupplementMix (PromoCell, Heidelberg, Germany).

Plasmids were transfected by calcium phosphate co-precipitation into cell lines, as described [11], or with fugene HD (Roche Diagnostics, Mannheim, Germany) into primary human cervical keratinocytes, following the manufacturer's protocol. Synthetic siRNAs were transfected with DharmaFECT (Dharmacon, Thermo Fisher Scientific, Waltham, MA) into HeLa cells or with Lipofectamine RNAimax (Invitrogen, Life Technologies) into SiHa cells at a final concentration of 10 nM, according to the manufacturer's protocol.

For DNA damage treatment with drugs, cells were plated on 6-cm dishes, transfected with siRNAs, and treated 24 h later with 1 µM camptothecin (CPT, Enzo Life Science, Lörrach, Germany) or 200 µg/ml hygromycin B (Invitrogen, cat. # 10687-010, LOT # HY064-L6, purity >90%, please refer to: http://tools.lifetechnologies.com/content/sfs/COAPDFS/2013/HY064-L6_10687010.pdf), for the indicated time periods. For ionizing radiation treatment, a Gammacell 1000 ^137^Cs source was employed (dose rate 5.85 Gy/min, Atomic Energy of Canada Ltd., Edmonton, Canada).

### Retroviral transduction of primary keratinocytes

Primary human foreskin keratinocytes (HFK) stably expressing HPV16 *E6* and/or *E7* were generated as previously described [70], by transduction with the retroviral vector pLXSN carrying either the HPV16 *E6* or *E7* open reading frames or the HPV16 *E6/E7* bicistronic sequence. As negative control, HFK were retro-transduced with empty vector pLXSN.

### Plasmids and synthetic siRNAs

siRNAs were either chemically synthesized (Ambion, Life Technologies) or expressed as shRNAs from vectors pCEPsh (selectable via its hygromycin B resistance) or pSUPER, as previously described [71]. The si/shRNA target sequences were as follows: 16E6-1 5′-ACCGUUGUGUGAUUUGUUA-3′, 16E6-2 5′-GGGAUUUAUGCAUAGUAUA-3′, 16E6-3 5′-UUAGUGAGUAUAGACAUUA-3′, 16E6/E7-1 5′-CCGGACAGAGCCCAUUACA-3′, 16E6/E7-2 5′-CACCUACAUUGCAUGAAUA-3′, 16E6/E7-3 5′-CAACUGAUCUCUACUGUUA-3′, 18E6-1 5′-GACAUUAUUCAGACUCTGU-3′, 18E6-2 5′-CAGACUCUGUGUAUGGAGA-3′, 18E6-3 5′-CUCUGUGUAUGGAGACACA-3′, 18E6/E7-1 5′-CCACAACGUCACACAAUGU-3′, 18E6/E7-2 5′-CAGAGAAACACAAGUAUAA-3′, 18E6/E7-3 5′-UCCAGCAGCUGUUUCUGAA-3′, LEDGF-1 5′-AGACAGCAUGAGGAAGCGA-3′ [72], LEDGF-2 5′-GGTAATCAGCCACAACATA-3′ [19], LEDGF-3 5′-GCAATGAGGATGTGACTAA-3′ [19]. The control si/shRNAs (Contr-1 5′-CAGUCGCGUUUGCGACUGG-3′ [73] and Neg 5′-UACGACCGGUCUAUCGUAG-3′ [74]) contain at least four mismatches to all known human genes.

For LEDGF reconstitution experiments, a *LEDGF* cDNA containing seven synonymous mutations in the shLEDGF-1 target site [75] was cloned into pCEP4 vector, yielding pLEDGF. A derivative of pLEDGF containing a point mutation in the PWWP-domain (pLEDGF-W21A) was generated by site-directed mutagenesis. Both wildtype LEDGF and LEDGF-W21A protein were Flag-tagged. The luciferase-reporter plasmid containing the *LEDGF/p75* promoter (pGL4.10LEDGFp75 −723/+59) was kindly provided by Drs. S. Desfarges and A. Ciuffi (University Hospital Center and University of Lausanne, Switzerland) [60]. HPV16, HPV18, HPV6 and HPV11 *E6* and *E7* expressing plasmids have been described previously [38], [71], [76].

### Luciferase assays

All luciferase assays were performed independently at least thrice, as double or triple values, following a previously described protocol [38]. In brief, cells were transfected with the *LEDGF* luciferase reporter plasmid, together with the indicated E6 or E7 expression vectors or pSUPER constructs. As an internal standard, each transfection also included a β-galactosidase reporter plasmid (pCMV-Gal) in order to correct for variations in transfection efficiencies [7]. Cells were harvested 48–72 h post transfection and processed as described [7]. Luciferase activities were quantified using a Lucy 1 microplate luminometer (Anthos, Krefeld, Germany).

### RNA extraction and quantitative reverse transcription polymerase chain reaction (qRT-PCR)

All qRT-PCR analyses were independently performed at least thrice, in duplicates. Total RNA from cells was isolated with the Pure Link RNA Mini Kit (Ambion, Life Technologies). Reverse transcription of 1 µg RNA was performed by using oligo-dT or random primers of the ProtoScript M-MuLV Taq RT-PCR Kit (New England Biolabs, Ipswich, MA), according to the manufacturer's instructions. For HFKs, total cellular RNA was extracted using the Absolutely RNA Miniprep kit (Agilent Technologies, Santa Clara, CA). One µg of RNA from each sample was reverse transcribed to cDNA by using RevertAid H Minus M-MuLV Reverse Transcriptase (MBI Fermentas, Thermo Fisher Scientific). Expression levels were determined by real-time PCR with a 7300 Real-Time PCR System detector (Applied Biosystems, Carlsbad, CA), using the SYBR green PCR Master Mix (Applied Biosystems). The forward (fwd) and reverse (rev) primer sequences (Eurofins MWG, Ebersberg, Germany) were as follows: 16E6 fwd 5′-AGCAATACAACAAACCGTTGTGT-3′, 16E6 rev 5′-CCGGTCCACCGACCCCTTAT-3′, 18E6 fwd 5′-AGACAGTATACCCCATGCTGCAT-3′, 18E6 rev 5′-TCCAATGTGTCTCCATACACAGA-3′, 18E6/E7 fwd 5′-ATGCATGGACCTAAGGCAAC-3′, 18E6/E7 rev 5′-AGGTCGTCTGCTGAGCTTTC-3′, LEDGF fwd 5′-CAAGGGAAGAAAGGGCCAAACA-3′, LEDGF rev 5′-CGTGCTGGCTTCATGGTTGT-3′, β-Actin fwd 5′-GGACTTCGAGCAAGAGATGGC-3′, β-Actin rev 5′-GCAGTGATCTCCTTCTGCATC-3′, GAPDH fwd 5′-GAAGGTGAAGGTCGGAGTC-3′, GAPDH rev 5′- GAAGATGGTGATGGGATTTC-3′, 18S RNA fwd 5′-CATGGCCGTTCTTAGTTGGT-3′, 18S RNA rev 5′-ATGCCAGAGTCTCGTTCGTT-3′. Cycling conditions have been described previously [77]. The sizes of the PCR products were initially verified by agarose gel electrophoresis and subsequently checked by melting point analysis after each reaction. Relative quantification was performed using the comparative Ct (2^−ΔΔCt^) method [78]. Data are presented as the fold difference in gene expression normalized to a reference gene (β-Actin, GAPDH or 18S RNA) and relative to a calibrator sample.

### Immunoblot analyses

All immunoblot experiments were performed at least three times, if not otherwise indicated. Total protein extracts were prepared 48–96 h after transfection. Cell pellets were lysed in CSK-1 buffer (10 nM Pipes pH 6.8, 300 mM NaCl, 1 m M EDTA, 300 mM Sucrose, 1 mM MgCl_2_, 0.5% TritonX-100), supplemented with Pefabloc (Merck, Whitehouse Station, NJ), Inhibitor Cocktail (Sigma-Aldrich) and phosphatase inhibitor cocktail (Roche Diagnostics) for 30 min on ice. Proteins were collected by centrifugation at 12,000 *g* for 10 min and protein concentrations were determined by the Bio-Rad Protein Assay (Bio-Rad, Hercules, CA). For Western blot analyses, protein extracts were separated on NuPAGE Novex 4–12% Bis-Tris Mini Gels (Life Technologies). Proteins were electrotransferred to an Immobilon-P membrane (Millipore, Bedford, MA), using the Trans-Blot Semi-Dry Transfer Cell (Bio-Rad). Membranes were blocked with 5% skim milk powder (Saliter, Obergrünzburg, Germany) and 1% bovine serum albumin (BSA, Sigma-Aldrich) in PBS-T (PBS/0.1% Tween-20) for 1 h at room temperature. Membranes were incubated with primary antibodies overnight at 4°C in PBS-T/5% skim milk powder/1% BSA, followed by incubation with the corresponding HRP-conjugated secondary antibody for 1 h at room temperature. Proteins were visualized using ECL Prime Western Blotting Detection Reagent (GE Healthcare, Buckinghamshire, UK). Images were monitored using Fusion SL Gel Detection System (Vilber Lourmat, Marne-la-Vallée, France). Band densities were determined by BioID image analysis software (Vilber Lourmat), relative to the respective loading controls.

The following primary antibodies were used: mouse anti-α-Tubulin (Merck), mouse anti-β-Actin (Sigma-Aldrich), chicken-anti-HPV18 E7 (E7C) [11], mouse-anti-p53 (BD Biosciences, Heidelberg, Germany), rabbit-anti-LEDGFp75 (Bethyl Laboratories, Montgomery, TX), rabbit-anti-Flag (Sigma-Aldrich) and mouse-anti-γH2AX (Ser139, Millipore). The following secondary HRP-conjugated antibodies were used: anti-mouse IgG (W3021, Promega, Madison, WI), anti-rabbit IgG (W4011, Promega), and anti-chicken IgY (G1351, Promega).

### Cell cycle analyses

For blocking HeLa cells in different cell cycle phases, cells were treated with 400 µM mimosine, 2 mM thymidine or 0.04 µg/ml nocodazole (all from Sigma-Aldrich), respectively, for 16 h. Cells were trypsinized, washed in ice-cold PBS and fixed in 70% cold ethanol overnight at −20°C. Subsequently cells were pelleted, resuspended in PBS containing 1 mg/ml RNase A (Roche Diagnostics) and 25 µg/ml propidium iodide (Sigma-Aldrich) and incubated for 30 min at room temperature. Cell cycle analyses were performed by fluorescence-activated cell sorting (FACS) using a FACSCalibur (BD Biosciences) with CellQuest Pro software provided by the manufacturer. Apoptotic cells were excluded and quantitation of the percentage of cells in the individual phases was performed using FlowJo software (Tree Star, Ashland, OR), applying the Watson model [79].

### Colony formation assays (CFAs)

For CFAs with pCEPsh vectors, cells were transfected and selected for hygromycin B resistance. Colonies were fixed and stained with formaldehyde-crystal violet, 10 to 13 days after transfection. For CFAs using synthetic siRNAs, cell numbers were determined with a Countess Cell Counter (Invitrogen) at 24 h post transfection. Cells were plated on 6-cm dishes (1,000 cells/dish) and treated the next day with 10 nM or 100 nM CPT for 1 h or with ionizing radiation (6 Gy). Colonies were fixed and stained with formaldehyde-crystal violet, 6 to 8 days following DNA damage treatment, and cell clones were counted.

### Immunohistochemistry

For validating anti-LEDGF antibody 6E4 (anti-PSIP1, Thermo Fisher Scientific; detects the LEDGF/p75 but not the LEDGF/p52 isoform), HeLa cells were plated on 6-cm dishes and transfected with either siLEDGF-1 or pLEDGF, or left untreated. Cells were trypsinized 72 h post transfection, washed in ice-cold PBS and pelleted. Cell pellets containing at least 2×10^6^ cells were suspended in a methanol-based preservation solution (PreservCyt, Hologic, Wiesbaden, Germany) and prepared as thin-layer cytology slides (ThinPrep, Hologic). Specimens were analyzed for LEDGF expression, using the staining protocol for immunohistochemistry detailed below.

For immunohistochemistry analyses, serial sections of formalin-fixed, paraffin-embedded cervical cancer cone biopsy specimens were dewaxed and rehydrated using xylene and a series of graded alcohols. Heat-induced antigen retrieval was performed by immersing the sections in a 10 mM citrate buffer solution (pH 6.0) and microwaving them for 3×5 min at 550 W. Slides were cooled in the antigen retrieval solution for 20 min. Endogenous peroxidase activity was blocked by incubating the sections in 1% hydrogen peroxide in methanol for 20 min at room temperature. Non-specific protein binding sites were blocked by incubating the slides in 10% horse serum diluted in PBS for 30 min. Sections were incubated over night at 4°C with primary antibodies diluted in PBS supplemented with 1% horse serum. The following primary antibodies were used: mouse-anti-p16^INK4a^ (CINtec Histology, Roche mtm Laboratories, Mannheim, Germany), mouse-anti-Ki67 (MIB-1, Dako, Hamburg, Germany), and mouse-anti-LEDGF (anti-PSIP1, 6E4, Thermo Fisher Scientific). Subsequent thorough washing in 0.1% PBS-T was performed. Sections were then incubated with a biotinylated secondary anti-mouse antibody (Vectastain Elite ABC, Vector Labs, Burlingame, CA) for 30 min at room temperature, followed by incubation with an avidin-biotin complex peroxidase (Vectastain Elite ABC) for 20 min at room temperature. LEDGF, p16 and Ki67 expression were visualized by a brown 3,3′-diaminobenzidine and abbreviation (DAB) reaction. Sections were glass-covered and analyzed by light microscopy (Olypmus Vanox-T, Hamburg, Germany) using a magnification up to ×400.

For immunohistochemical assessment of LEDGF expression, the product of the scores of staining frequency and intensity of immunoreactive cells was calculated as described [80]: the frequency ranged from 5% to 100% of LEDGF-positive cells, and the intensity comprised 1 = low to 3 = high. The final immunohistochemical score (ranging from 5 to 300) was obtained by multiplication of the intensity score and the frequency score. All sections were independently reviewed in random order by two researchers (JL and MR). For the few instances of discrepant scoring, a consensus score was determined. Formalin-fixed, paraffin-embedded tissue blocks were used anonymized without linked personal data according to the regional ethical regulations.

### Statistical analyses

Statistical significance of differences in measured variables between controls and treated samples was evaluated by a two-sided paired t-test using the Sigma Plot software (Systat Software Inc., San Jose, CA). For immunohistochemical analyses statistical significance of differences in calculated scores between histologically normal and HPV-positive samples was determined by two-sided t-test using the SPSS software version 21 (Armonk, NY: IBM Corp.). p-values of ≤0.05 (*), ≤0.01 (**), or ≤0.001 (***) were considered statistically significant.

### Accession numbers

Genebank accession numbers according to the National Center for Biotechnology Information (http://www.ncbi.nlm.nih.gov/genbank) for genes and proteins discussed in this paper are as follows: *PSIP1* (Gene ID 11168), LEDGF (NP_150091.2), *HPV16 E6* (Gene ID 1489078), HVP16 E6 (NP_041325.1), *HPV16 E7* (Gene ID 1489079), HPV16 E7 (NP_041326.1), *H2AX* (Gene ID 3014), H2AX (NP_002096.1).

## Supporting Information

Figure S1 Validation of anti-LEDGF antibody 6E4.
(PDF)

Table S1 LEDGF expression in cervical tissue.
(DOC)
