# Supplementary material for: Oncogenic Human Papillomaviruses Activate the Tumor-Associated Lens Epithelial-Derived Growth Factor (LEDGF) Gene
Source: PLoS Pathog. 2014 Mar 6;10(3):e1003957. doi: 10.1371/journal.ppat.1003957 (PMC3946365; doi:10.1371/journal.ppat.1003957)
Supplement: Figure S1 — Validation of anti-LEDGF antibody 6E4. (PDF) [file ppat.1003957.s001.pdf]

Supplemental Figure S1.

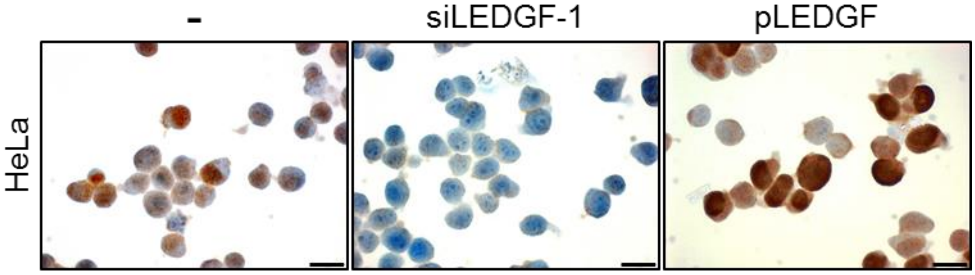

Validation of anti-LEDGF antibody 6E4. Untreated HeLa cells (-), HeLa cells transfected siLEDGF-1, and HeLa cells overexpressing LEDGF from pLEDGF were prepared on thin-layer cytology slides and stained for LEDGF expression. Bars correspond to 20 µm.
