# Supplementary material for: Oncogenic Human Papillomaviruses Activate the Tumor-Associated Lens Epithelial-Derived Growth Factor (LEDGF) Gene
Source: PLoS Pathog. 2014 Mar 6;10(3):e1003957. doi: 10.1371/journal.ppat.1003957 (PMC3946365; doi:10.1371/journal.ppat.1003957)
Supplement: Table S1 — LEDGF expression in cervical tissue. (DOC) [file ppat.1003957.s002.doc]

**Table S1. LEDGF expression in cervical tissue.**

|  |  | **normal** | **CIN I** | **CIN II** | **CIN III** | **SCC** |
| --- | --- | --- | --- | --- | --- | --- |
| **frequency^a^** | range [%] | 5-40 | 5-80 | 5-95 | 50-95 | 20-100 |
|  | 25% quartile | 5,5 | 15 | 10 | 75 | 30 |
|  | median | 8 | 20 | 80 | 95 | 80 |
|  | 75% quartile | 13.75 | 40 | 95 | 95 | 95 |
| **intensity^b^** | 1 | 17/36 (47.2%) | 4/16 (25.0%) | 1/7 (14.3%) | 0/13 (0%) | 0/7 (0%) |
|  | 2 | 18/36 (50.0%) | 12/16 (75.0%) | 5/7 (71.4%) | 4/13 (30.8%) | 2/7 (28.6%) |
|  | 3 | 1/36 (2.8%) | 0/16 (0%) | 1/7 (14.3%) | 9/13 (69.2%) | 5/7 (71.4%) |
| **score^c^** | range [%] | 5-80 | 5-160 | 5-285 | 100-285 | 60-300 |
|  | 25% quartile | 5.5 | 16.25 | 20 | 155 | 60 |
|  | median | 15 | 40 | 160 | 285 | 240 |
|  | 75% quartile | 20 | 80 | 190 | 285 | 285 |

^a^percentage positive cells, ^b^ increasing intensity from 1= low to 3 = high, ^c^score = product of frequency and intensity
